# Opportunities for Web-based Drug Repositioning: Searching for Potential Antihypertensive Agents with Hypotension Adverse Events

**DOI:** 10.2196/jmir.4541

**Published:** 2016-04-01

**Authors:** Kejian Wang, Mei Wan, Rui-Sheng Wang, Zuquan Weng

**Affiliations:** ^1^CoMed Technology & Consulting Co., Ltd.Hong KongChina; ^2^College of Biological Science and EngineeringFuzhou UniversityFuzhouChina; ^3^Japan National Institute of Occupational Safety and HealthKawasakiJapan

**Keywords:** Web-based drug repositioning, FDA Adverse Event Reporting System, FAERS, openFDA, big data, antihypertensive drugs, hypotension

## Abstract

**Background:**

Drug repositioning refers to the process of developing new indications for existing drugs. As a phenotypic indicator of drug response in humans, clinical side effects may provide straightforward signals and unique opportunities for drug repositioning.

**Objective:**

We aimed to identify drugs frequently associated with hypotension adverse reactions (ie, the opposite condition of hypertension), which could be potential candidates as antihypertensive agents.

**Methods:**

We systematically searched the electronic records of the US Food and Drug Administration (FDA) Adverse Event Reporting System (FAERS) through the openFDA platform to assess the association between hypotension incidence and antihypertensive therapeutic effect regarding a list of 683 drugs.

**Results:**

Statistical analysis of FAERS data demonstrated that those drugs frequently co-occurring with hypotension events were more likely to have antihypertensive activity. Ranked by the statistical significance of frequent hypotension reporting, the well-known antihypertensive drugs were effectively distinguished from others (with an area under the receiver operating characteristic curve > 0.80 and a normalized discounted cumulative gain of 0.77). In addition, we found a series of antihypertensive agents (particularly drugs originally developed for treating nervous system diseases) among the drugs with top significant reporting, suggesting the good potential of Web-based and data-driven drug repositioning.

**Conclusions:**

We found several candidate agents among the hypotension-related drugs on our list that may be redirected for lowering blood pressure. More important, we showed that a pharmacovigilance system could alternatively be used to identify antihypertensive agents and sustainably create opportunities for drug repositioning.

## Introduction

Drug repositioning, also referred to as drug repurposing, is the process of developing new indications for existing drugs [1]. The financial advantage of drug repositioning over traditional drug development is that much of the cost and time spent in the early stage can be bypassed. In addition, the risk of failure caused by adverse reactions can be better controlled, since the toxicity of the repurposed drugs has already been tested [2]. For these reasons, both the pharmaceutical industry and academic communities are paying particular attention to this field. An increasing number of *in silico* [3,4] and *in vitro* [5-7] approaches have been developed to efficiently scan the existing pharmacopoeia for new usage. Other than these strategies primarily focused on preclinical information, side effects data are increasingly used for rational drug repositioning [8-10], due to the direct reflection of the clinical reality of actual patients [11]. The collection of side effects information based on clinical trials is conventionally a time-consuming and labor-intensive process. Making things more difficult, most raw data are not freely available to the public. However, the big data concept and Internet-related technologies are making it easier to access and analyze side effects records.

Hypertension, characterized by aberrantly high arterial blood pressure, is a chronic medical condition affecting almost one billion people worldwide [12]. Despite the availability of several blood pressure-decreasing drugs, hypertension is not effectively controlled in more than half of patients receiving antihypertensive treatment [13]. Among the multiple reasons contributing to this unsatisfactory clinical outcome, an undeniable reality is that most pharmaceutical companies, considering the potential costs and profits, have abandoned antihypertensive drug development [14]. As drug pipelines dry up, new drug development is expensive and time consuming, thus diminishing future profitability [15]. Worse still, the high failure rate (estimated to be >90%) due to toxicity and other reasons has made new drug development a highly risky investment [16]. Therefore, alternative strategies are urgently needed to drive the productivity and cost-effectiveness of antihypertensive drug development. Assuming that hypotension, with the symptom of abnormally low blood pressure, can be regarded as the opposite condition of hypertension, then potential antihypertensive agents may be discovered among drugs that induce hypotension as a side effect.

Established by the US Food and Drug Administration (FDA), the FDA Adverse Event Reporting System (FAERS) [17] is one of the most comprehensive sources of pharmacovigilance big data worldwide. With adverse drug events spontaneously submitted by consumers and indirectly reported by health care professionals, FAERS supports not only FDA’s safety surveillance on all approved drugs, but also the research of scientists and clinicians. Each report provides a variety of clinical information, particularly the drug(s) used by the patient and the adverse reaction(s) that the patient experienced. FAERS’ raw data used to be released without sufficient formatting, thus making a high throughput analysis very difficult. Fortunately, the FDA’s Office of Informatics and Technology Innovation launched a new initiative, OpenFDA, in 2013 [18,19], whose primary goal was to facilitate public access to high-value FDA data (including FAERS) by providing user-friendly and open-source application programming interfaces.

**Figure 1:**
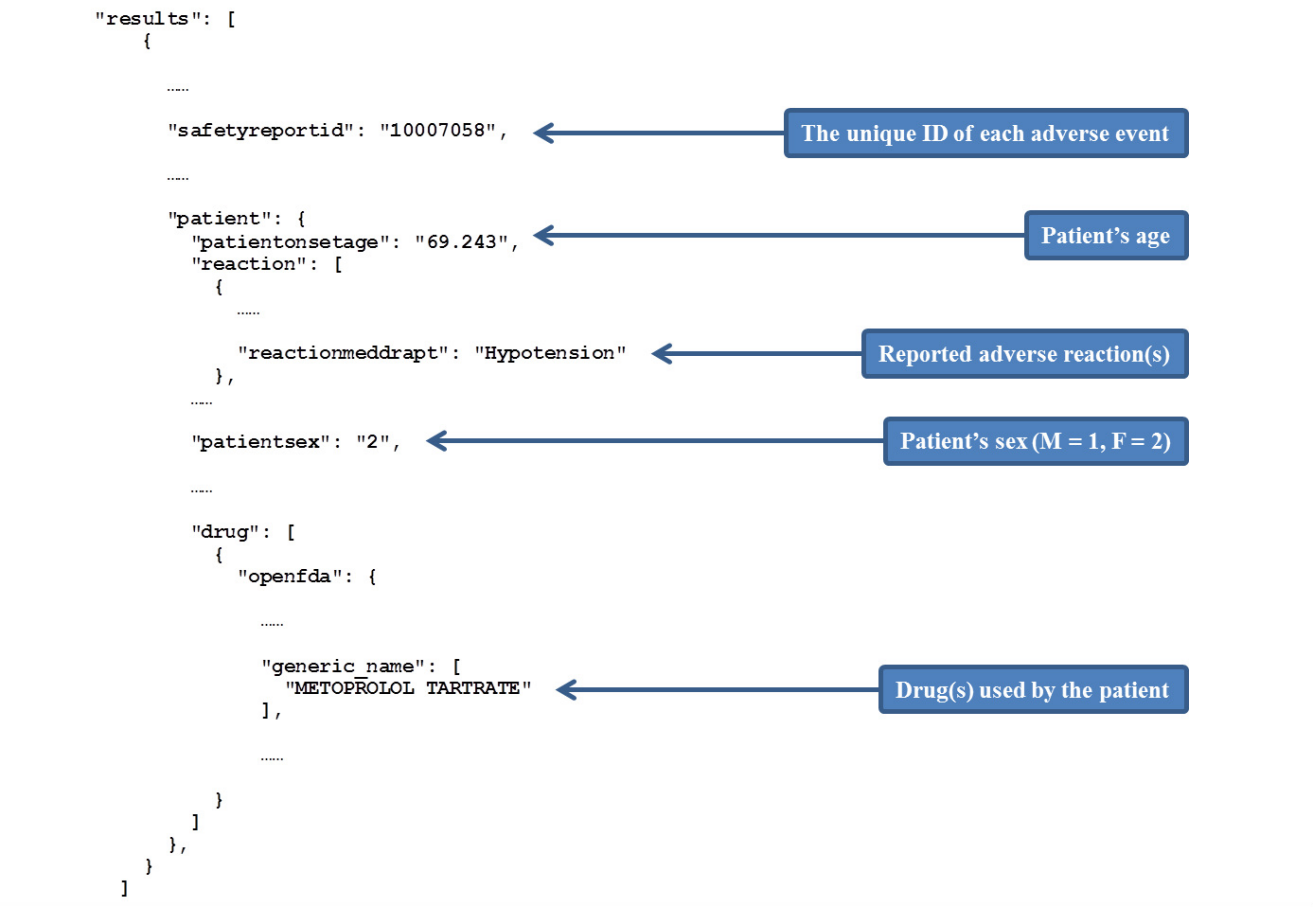
Co-occurrence of drug and adverse reaction. An adverse event with unique identifier (ID) can be queried in the US Food and Drug Administration (FDA) Adverse Event Reporting System (FAERS), which returns results as JavaScript Object Notation by default. In this illustrated adverse event (with partial content displayed), the use of metoprolol and the incidence of hypotension reaction are reported simultaneously, which is defined as co-occurrence.

By using the OpenFDA platform and relevant statistical methods [20], we aimed to examine the co-occurrence of specific drugs and hypotension as a side effect, that is, the drug and a hypotension reaction co-occurred in the same adverse event report (Figure 1). Instead of serendipitous searching, we examined the correlation between hypotension incidence and antihypertensive property for a total of 683 unique drugs, so as to identify those that frequently co-occurred with hypotension adverse events. As an exploratory effort in systematically analyzing drug adverse events, we hoped that this study would provide a unique insight into Web-based and data-driven drug repositioning.

## Methods

### FAERS Data Query

We retrieved the raw FAERS reports (from January 2004 to September 2013) from the public adverse events dataset of OpenFDA in December 2014, according to the official tutorial on query tools [9]. We primarily investigated the 1000 most commonly reported generic names. By merging multiple generic names corresponding to the same drug product (eg, “abacavir” and “abacavir sulfate”) and excluding combinations of multiple active ingredients (eg, “oxycodone and acetaminophen”), we identified a total of 683 unique drugs. For each drug, we searched for hypotension-related events using the terms “hypotension,” “blood pressure decreased,” and “orthostatic hypotension.”

### Statistical Analysis

For each drug of interest, we constructed a 2×2 contingency table to serve as the framework for analysis of all FAERS reports. For the reports with adverse event co-occurrence with the drug, we defined the numbers of events including hypotension as n11 and excluding hypotension as n10. For the reports not related to the drug of interest, we defined the numbers of events with hypotension as n01 and without hypotension as n00. Then, we calculated the hypotension reporting odds ratio (ROR) as (n11 × n00) / (n10 × n01). We determined the statistical significance of the ROR by Fisher exact test.

### Normalized Discounted Cumulative Gain

**Figure 2:**
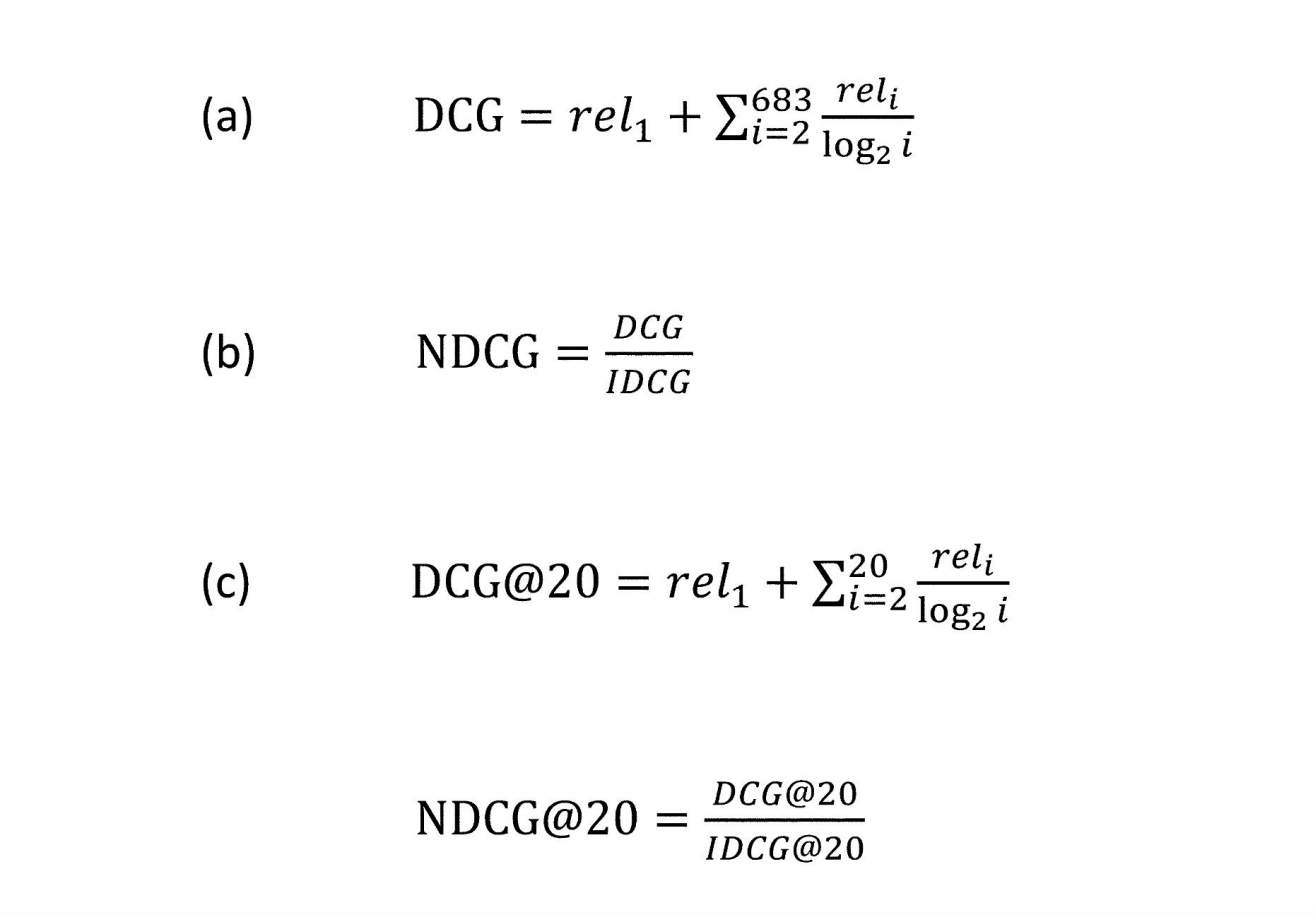
Equations for calculating crude discounted cumulative gain (DCG), normalized discounted cumulative gain (NDCG), and NDCG for the top 20 drugs (NDCG@20). DCG@20: DCG of the top 20 drugs; IDCG: ideal discounted cumulative gain; IDCG@20: IDCG of the top 20 drugs; rel_1_: relevance score for approved antihypertensive drugs; rel_i_: relevance score.

Normalized discounted cumulative gain (NDCG) measures the relevance of a document based on its position in the result list. The gain is accumulated from the top of the result list to the bottom, with the gain of each result discounted at lower ranks. We ranked each of the 683 drugs by the significance of its ROR and judged it on a relevance score, with 1 meaning approved antihypertensives and 0 meaning other, irrelevant drugs. We calculated the crude discounted cumulative gain (DCG) by equation (a) in Figure 2.

To normalize the DCG value, we produced an ideal ordering for the 683 drugs (the ranking with maximum possible DCG) to calculate the ideal discounted cumulative gain. Then the NDCG, ranging from 0 to 1, was calculated by equation (b) in Figure 2. The closer the NDGC value is to 1, the better the performance. In addition, we calculated the NDCG for the top 20 drugs (NDCG@20) by equation (c) in Figure 2. Similarly, the closer the NDGC@20 value is to 1, the better the performance in terms of identifying the top 20 drugs.

## Results

### Co-occurrence of Well-Known Antihypertensive Drugs and Hypotension Reports

To justify the basic assumption that hypotension events may suggest antihypertensive activity, we expected the approved antihypertensive drugs to be reported more often for hypotension. To achieve broad drug coverage, we examined 683 study drugs with a single active ingredient for hypotension adverse events (Multimedia Appendix 1). As was done previously in a series of FAERS-based studies [21,22], we calculated the significance level of the ROR [23] to assess the association between drug use and hypotension incidence, according to the numbers of hypotension and nonhypotension reports that co-occurred with the drug of interest (see Methods).

**Figure 3:**
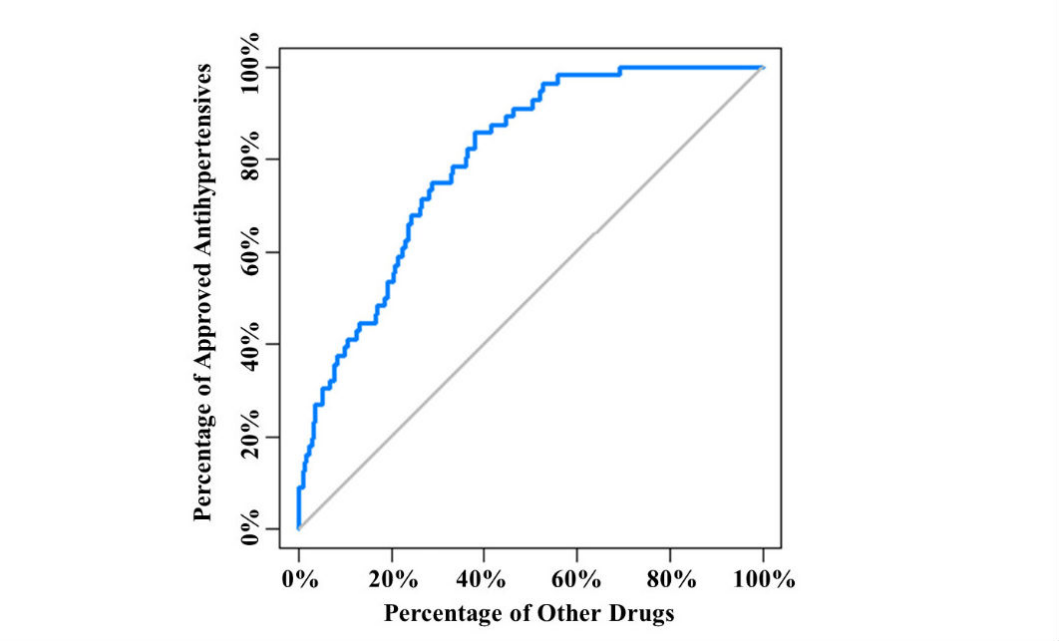
Correlation between antihypertensive activity and hypotension reporting in the US Food and Drug Administration (FDA) Adverse Event Reporting System (FAERS), showing the classification between approved antihypertensives and other drugs.

**Figure 4:**
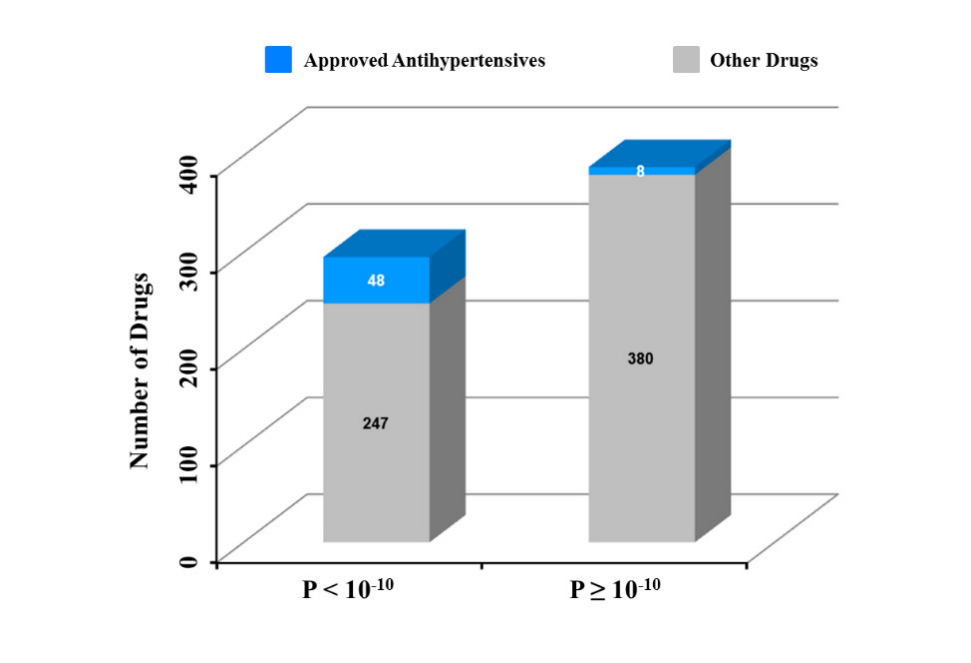
Correlation between antihypertensive activity and hypotension reporting in the US Food and Drug Administration (FDA) Adverse Event Reporting System (FAERS), showing the number of drugs with significant hypotension reporting with regard to the cut-off of *P*<10^-10^.

We ranked all of the 683 study drugs by the significance level (in terms of unadjusted *P* value), which pinpointed well-known FDA-approved antihypertensives according to drug indication information that we retrieved from DrugBank [24]. We found that the approved antihypertensives were effectively distinguished from other study drugs. The approved antihypertensives were highly represented among the top significant drugs (ie, with the lowest *P* values), and the area under the receiver operating characteristic (ROC) curve was >0.80 (Figure 3). When the percentage of nonantihypertensive drugs (ie, false-positive rate) was <10%, the proportion of approved antihypertensives was nearly 40% and the partial area under the ROC curve was 0.026. In addition, we calculated the NDCG [25-29] to measure the quality of the ranking of approved antihypertensives (see Methods). The overall NDCG and NDCG@20 of our model were 0.77 and 0.59, respectively, showing a remarkable performance. Among the approved antihypertensives, 86% (48/56) achieved an unadjusted *P*<10^–10^. On the other hand, the percentage for other drugs was only 39.4% (247/627). Such a dramatic difference (Figure 4) (odds ratio 9.21, Fisher exact test *P*=8.96×10^–12^) indicated that hypotension reporting in FAERS could serve as a phenotypic indicator and effectively reflect the well-known antihypertensive activity.

### Frequent Hypotension Reporting as an Indicator of Potential Antihypertensive Agents

Since the correlation has been proved between the reporting of hypotension as a side effect and the antihypertensive indication, we speculated that the drugs still not approved for treatment of hypertension but frequently co-occurring with hypotension adverse events might lead to hidden opportunities for drug repositioning. To test this hypothesis, we preliminarily surveyed all of the top 20 drugs as ranked by the significance level (Table 1). 

Among the top 11 drugs with a *P* value close to zero, 5 are approved antihypertensive drugs: metoprolol, spironolactone, furosemide, lisinopril, and carvedilol. Surprisingly, most of the other top-ranked drugs were also reported for hypotensive effects, particularly in humans. Aspirin, in addition to its importance as a widely used analgesic and anti-inflammatory drug, has been repeatedly reported to improve blood pressure control in hypertensive patients [30-32]. Digoxin, originally indicated for arrhythmias and heart diseases, was also found in various double-blind studies to significantly decrease diastolic blood pressure during overnight sleep [33,34]. As an essential mineral in the body, potassium has been clinically proven to lower blood pressure in humans [35-37]. And according to the package inserts (ie, the warnings and precautions subsection), as approved by the FDA, the anesthetic drugs morphine [38] and propofol [39] have a warning for inducing hypotension in clinical trials. This leads to the conclusion that FAERS can be efficiently screened for additional molecules, besides the approved antihypertensive drugs, with potential antihypertensive properties of the additional molecules supported by independent clinical evidence.

**Table 1:** The top 20 drugs that most frequently co-occurred with hypotension adverse events.

Drug	ATC^a^ code	*P* value	ROR^b^	Adverse events co-occurring with drug (n)		Adverse events not co-occurring with the drug (n)	
				Hypotension	Not hypotension	Hypotension	Not hypotension
Metoprolol	C07AB02^c^	0	2.99	3287	62,998	64,291	3,683,647
Spironolactone	C03DA01^c^	0	3.71	1581	24,021	65,997	3,722,624
Furosemide	C03CA01^c^	0	3.49	5173	86,868	62,405	3,659,777
Lisinopril	C09AA03^c^	0	2.69	3465	73,779	64,113	3,672,866
Carvedilol	C07AG02^c^	0	4.11	1955	26,954	65,623	3,719,691
Propofol	N01AX10	0	7.81	1098	7903	66,480	3,738,742
Digoxin	C01AA05	0	4.44	2259	28,945	65,319	3,717,700
Potassium	N/A	0	3.51	1533	24,635	66,045	3,722,010
Morphine	N02AA01	0	3.02	1683	31,375	65,895	3,715,270
Warfarin	B01AA03	0	2.52	3076	69,491	64,502	3,677,154
Aspirin	A01AD05 B01AC06 N02BA01	0	2.46	6107	145,276	61,471	3,601,369
Amlodipine	C08CA01^c^	3.95E–315	2.29	2854	70,799	64,724	3,675,846
Isosorbide	C01DA14^c^	5.12E–319	4.16	1139	15,390	66,439	3,731,255
Clopidogrel	B01AC04	9.83E–306	2.45	2395	55,283	65,183	3,691,362
Acetaminophen	N02BE01	8.37E–302	2.51	2266	51,149	65,312	3,695,496
Atenolol	C07AB03^c^	4.26E–298	2.52	2223	49,993	65,355	3,696,652
Midazolam	N05CD08	1.30E–296	7.13	641	5023	66,937	3,741,622
Ondansetron	A04AA01	9.23E–295	3.51	1293	20,700	66,285	3,725,945
Lorazepam	N05BA06	3.13E–284	2.62	1965	42,386	65,613	3,704,259
Ramipril	C09AA05^c^	2.90E–275	2.97	1539	29,213	66,039	3,717,432

^a^ATC: Anatomical Therapeutic Chemical Classification System.

^b^ROR: reporting odds ratio.

^c^Approved antihypertensive drug.

In addition, we observed a possible correlation between the Anatomical Therapeutic Chemical (ATC) Classification System code and antihypertensive activity in the top 20 agents. As Table 1 shows, except for drugs designed for cardiac therapy (ie, indicated by the ATC first-level code C), other candidate agents were originally developed for treating the alimentary tract and metabolism (ie, ATC first-level code A), blood and blood-forming organs (ie, ATC first-level code B), or the nervous system (ie, ATC first-level code N). In particular, drugs acting on the nervous system were well represented in the top-ranked drugs, as evidence by a relatively high NDCG@20 value of 0.35 (Table 2). This observation was consistent with the commonly known interplay between blood pressure and the nervous system [40], suggesting that the hypotensive effects of nervous system agents can be detected effectively with patient-centric pharmacovigilance data. Therefore, along the direction of this study, particular attention may be paid to nervous system agents in further searches for novel antihypertensives.

**Table 2:** Noncardiac drug classes highly represented in the top 20 drugs.

ATC^a^ code (first level)	Drug class	Ranks in top 20	NDCG@20^b^
N	Nervous system	6, 9, 11, 15, 17, 19	0.35
B	Blood and blood-forming organs	10, 11, 14	0.17
A	Alimentary tract and metabolism	11, 18	0.12

^a^ATC: Anatomical Therapeutic Chemical Classification System.

^b^NDCG@20: normalized discounted cumulative gain for the top 20 drugs.

## Discussion

In recent years, conventional target-based drug development has been facing various challenges, such as increasingly higher costs and lower productivity. In consequence, both the pharmaceutical industry and the academic community are earnestly seeking new means of drug innovation. In this process, many realize that, along with improvements in clinical informatics, the role of patients can be changed from a minimally informed recipient to a potential participant in drug development [41,42]. By providing raw download access to structured datasets, OpenFDA has created a researcher-friendly portal for quick and easy querying. With patient-centric data being a highly cost-effective and valuable resource, we can make this systematic effort and corroborate the evidence linking hypotension as a side effect to an antihypertensive therapeutic effect.

For hypertension and many other medical conditions, rationally finding unknown therapeutic agents has always been a valuable but difficult task. Even though various methods are established to accelerate the process of selecting repositioning candidates, there remains at least one major barrier between theoretical therapeutic effects and real benefits. Due to the complexity of the human body, many repositioning approaches that are focused on omics and preclinical data may not always be consistent with patients’ clinical outcomes [43]. Regarding this issue, the unique advantage of clinical informatics analysis using FAERS should be appreciable. Basically, hypotension adverse events are observations on humans, as opposed to molecular entities, cell cultures, or animal models. The antihypertensive signals suggested by FAERS tend to be more interpretable and straightforward, presenting relatively fewer challenges to bench-to-bed translation.

Pharmacovigilance data are not the only source of side effect information, since package inserts (also known as drug labels) determined by regulatory agencies have also been studied for drug repositioning [10,44,45]. However, in several ways, our study suggests that the patient-based FAERS data play an irreplaceable role in side effect analysis. As the basis of drug labeling, clinical trials are mostly conducted among a relatively small number of people and over a limited period of time. In contrast, postmarketing drug surveillance is a long-term effort that involves the general population. Therefore, the incidence of various side effects can be more comprehensively monitored through the patients’ self-reports. In addition, drug labels usually focus on the existence of certain side effects, while the prevalence is mentioned less often. However, the frequency of hypotension is an essential parameter for estimating the size of the applicable population, which is directly related to the potential profitability of the repositioned drug. In this regard, the FAERS data enabled us to calculate the significance level of the hypotension ROR for all of the study drugs, efficiently screening for the promising candidates.

Despite the richness of the information, some limitations related to the voluntary nature of FAERS merit additional attention. Since the details about the patients’ medical history and the context of adverse events may not be sufficiently addressed in many spontaneously submitted reports, the co-occurrence of a specific drug and a side effect may not be directly interpreted as a cause-and-effect relationship [46]. To address this issue, the patient-expert relationship needs to be enhanced following the concept of participatory medicine [47,48]. For instance, an Internet-based community can be established to connect patients and experts. The first-hand information reported by patients can then be promptly scrutinized and curated by clinical experts, thus improving the reliability of the raw data. Beyond being a source of information, patients can therefore play a more proactive role in clinical informatics research.

Given the lack of a novel candidate molecule, as well as a new mechanism of action, in antihypertensive drug development for a long time now, our work may have both realistic and long-term implications. First, our results provide a collection of candidate agents that may decrease blood pressure. Since most of the top frequently reported drugs for hypotension events are either well-known antihypertensive drugs or were proved later to show antihypertensive activity, there are grounds for believing that novel antihypertensive agents can be found among other study drugs that we identified (see Multimedia Appendix 1). We therefore suggest that the highly significant signals of certain drugs should be followed up by experimental or clinical investigations. Second, and more important, this study has shown a sustainable way of detecting potential antihypertensive agents. As numerous adverse events are consistently submitted to FAERS and periodically released through the OpenFDA platform, not only the marketed drugs addressed in this study, but also new drugs approved in the future may be highlighted for subsequent hypotension adverse events. Third, we believe the rationale of the opposite condition, linking the hypotension side effect and hypertension treatment, can be expanded to other indications with elaborate study designs. As long as FAERS continues to be updated, more opportunities for drug repositioning may be persistently discovered. Eventually, the newfound agents and the underlying mechanism of action would promote the discovery and development of various therapies.

## Multimedia Appendix 1

Supplementary data regarding all 683 study drugs.

## Abbreviations

ATC: Anatomical Therapeutic Chemical Classification System
DCG: crude discounted cumulative gain
FAERS: FDA Adverse Event Reporting System
FDA: US Food and Drug Administration
NDCG: normalized discounted cumulative gain
NDCG@20: normalized discounted cumulative gain for the top 20 drugs
ROC: receiver operating characteristic
ROR: reporting odds ratio
